# Giant Gallbladder Presenting as a Right Iliac Fossa Mass Removed by Mini-laparoscopic Cholecystectomy

**DOI:** 10.7759/cureus.5576

**Published:** 2019-09-05

**Authors:** Joshua Fultang, Ugochukwu Chinaka, Abdulmajid Ali

**Affiliations:** 1 General Surgery, University Hospital Ayr/University of West of Scotland, Ayr, GBR; 2 General Surgery, University Hospital Ayr/University of West of Scotland, AYR, GBR; 3 General Surgery, University Hospital Ayr/University of West of Scotland, Ayr, GBR

**Keywords:** gallbladder, gallstones, laparoscopic cholecystectomy, mini laparoscopic cholecystectomy, giant gallbladder

## Abstract

Giant gallbladder (GGB) is a rare condition that can result from cholelithiasis or chronic cholecystitis. Although there are no clear-cut definitions, gallbladders of >14 cm and ≥1.5 L have been regarded as GGBs. To date, most GGBs have been managed by laparotomic removal. This report describes a patient with a GGB that presented as a right iliac fossa mass. The GGB was successfully removed by mini-laparoscopic cholecystectomy. A 63-year-old woman presented with painful swelling in her right lower abdomen associated with dyspepsia and a palpable right iliac fossa mass. Computed tomography of the abdomen revealed a markedly enlarged gall bladder (19.5 x 5.4 x 5.6 cm) containing stones. Magnetic resonance cholangiopancreatography ruled out extra- and intrahepatic ductal dilatation and stones. She underwent a mini-laparoscopic cholecystectomy, and her postoperative recovery was uneventful.

## Introduction

Giant gallbladder (GGB) is a rare condition, and as of 2014, only eight cases have been reported in the literature since the 18th century [1,2]. A case report in 2013 described details, including gallbladder dimension in four patients with GGBs [2]. Attempts have been made to define GGBs by weight, volume, and shape. Most patients to date have undergone laparotomy for removal of GGBs [3]. This report describes a patient with a GGB presenting as a right iliac fossa mass. The GGB was successfully removed by mini-laparoscopic cholecystectomy.

## Case presentation

**Figure 1:**
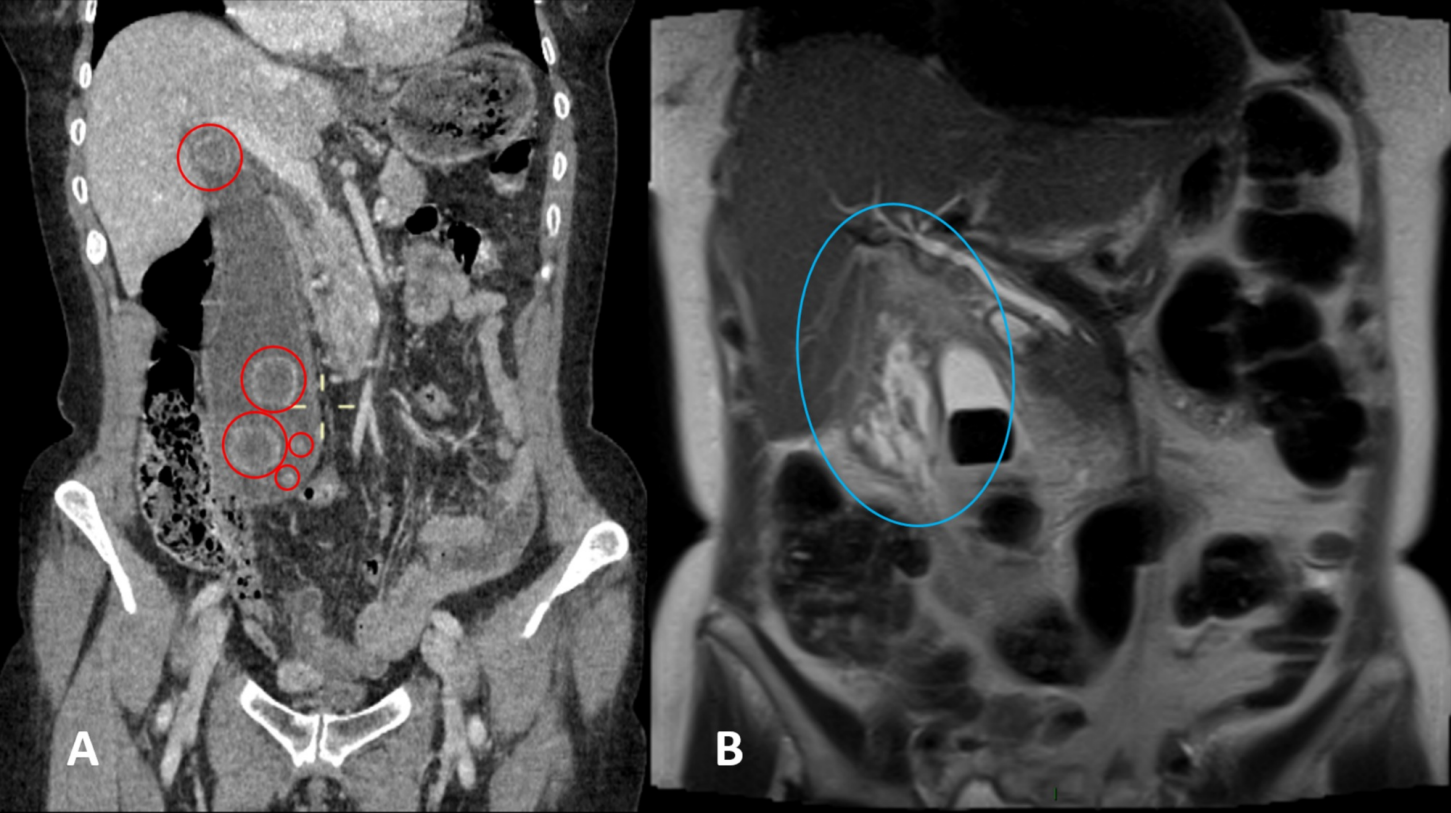
Abdominal computed tomography (CT) scan Abdominal computed tomography showing a markedly enlarged gallbladder containing several stones measuring (red circles) 19.5 x 5.4 x 5.6 cm in the (A) craniocaudal, transverse and anteroposterior dimensions and in the (B) anatomical pelvis. Significant edematous changes in the area between the distended gallbladder with stones and the liver suggested gallbladder perforation (blue circle).

A 63-year-old woman presented with a two-day history of right-sided abdominal pain and swelling associated with dyspepsia. She had no history of jaundice. Abdominal examination revealed abdominal fullness on her right side with a palpable right iliac fossa mass. She was admitted for further examination. Blood tests showed high concentrations of inflammatory markers. She experienced an episode of pyrexia, requiring the commencement of intravenous (IV) antibiotics. A computed tomography (CT) scan of the abdomen demonstrated a markedly enlarged gallbladder (Figure 1). She was subsequently readmitted for an elective mini-laparoscopic cholecystectomy which was successful. Her postoperative recovery was unevenly and she was discharged at 24 hours postoperatively. A review at six weeks after discharge in the outpatient general surgery clinic was satisfactory.

The removed gallbladder weighed 73.8 g. Histologic examination showed an accumulation of hemosiderophages within the wall mixed with foreign body type multinucleated giant cells, but no evidence of malignancy. The largest stone was cuboid and 27 mm in diameter.

## Discussion

GGBs are very rare, with few such cases reported to date (Tables 1, 2). There are no clear-cut parameters for differentiating a large gallbladder from a GGB [1,2]. Normal gallbladders are 7.5 to 10 cm in diameter, whereas some reported GGBs are >14 cm [2]. Gallbladders ≥1.5 L (similar to or larger than an adult liver) have been defined as GGBs [2].

**Table 1:** Case reports describing patients with GGB, including GB size and volume GB: Gallbladder; GGB: Giant gallbladder; F: Female; M: Male; NR: Not recorded.

Case report	Sex	Age (years)	Major comorbidity	GB size (cm)	GB volume	Patient cystic duct
Petit, before 1750 [4]	F	27-28	NR	NR	“2 pintes” (about 2 L)	Probable
Van Swieten, 1754 [5]	M	12	Very probable	NR	“8 libras” (about 2.6 L)	Yes
Collinson [6]	NR	NR	NR	NR	12.5 L	NR
Neudörfer, 1911 [6]	F	50	NR	NR	5.25 L	Yes
Kehr, 1913 [6]	NR	NR	NR	NR	1.5 L	NR
Borodach et al., 2005 [7]	F	67	NR	20 x 12	1.5 L	Yes
Panaro et al., 2012 [8]	NR	17	PFIC-2	43 x 21	2.7 L	Yes
Zong et al., 2013 [2]	F	55	NR	30 x 18	4.0 L	Yes
This case	F	77	NR	24 x 17	3.3 L	Yes

**Table 2:** Overview of reported cases of GGB GGB: Giant gallbladder; F: Female; M: Male; NR: Not recorded.

Case report	Sex	Age (years)	Size (cm)	Obstruction	Postoperative diagnosis
Grosberg, 1962 [9]	F	95	14 × 5.5	Stone	Acute gangrenous cholecystitis, cholelithiasis
Maeda et al., 1979 [10]	F	36	18 × 4	No	Chronic cholecystitis, cholelithiasis
Hsu et al., 2011 [11]	F	87	16.4 × 13.6 × 7.8	No	Acute cholecystitis, gall bladder adenocarcinoma
Panaro et al., 2013 [12]	NR	17	43 × 21 × 20	No	Byler’s disease

A GGB may present initially as a cyst or tumor in the abdominal cavity, a finding usually atypical of gall bladder diseases [1]. The finding of a palpable right iliac fossa mass, as in the present patient, may elude the diagnosis of GGBs. Short-term intraluminal hypertension due to a tumor, such as a pancreatic tumor, obstructing the biliary tract, may cause a gallbladder to become enlarged [3,12]. In contrast, gallstones, which form over an extended period, may result in a shrunken, fibrotic gall bladder [1,13].

GGBs may also be due to chronic obstruction, especially in patients with progressive conditions like malignancy [14]. This obstruction can induce chronically elevated intraductal pressure capable of producing an enlarged gallbladder. Stones can also cause intermittent obstructions, but these are regarded as not consistent enough to generate the chronic rise in intraductal pressure [14]. Chronic inflammation from stones may also result in the attenuation of the contractile function of the gallbladder, leading to further enlargement of an already distended gall bladder [15]. Acute blockage by migrating stones at the hepatic/cystic junction may also induce the growth of large gallbladders through a valve-like mechanism [2].

In addition to these obstructive mechanisms, GGBs may have other causes, including local hypoganglionosis within the gallbladder neck and other conditions allowing for the progressive enlargement of the gallbladder without clinical complications [1]. This patient reported in this study had gallstones.

A mini-laparoscopic cholecystectomy involves the use of small trocars and instruments, ranging from 2 mm to 3.5 mm, similar to those used for conventional laparoscopic cholecystectomy. The operation in this patient used 3-mm trocars. Successful mini-laparoscopic cholecystectomy requires surgical experience and adaptation, more in patients with unusually sized gallbladders. The umbilical port served as a good route for extraction following an extension of this port by a few centimeters.

## Conclusions

GGBs are rare, and their exact etiology and pathophysiology remain largely undetermined. No consensus has been reached on standardized definitions. GGBs can present as right iliac fossa masses containing multiple gallstones. Mini-laparoscopic cholecystectomy is effective and safe when performed by experienced surgeons.
